# Elective Neck Dissection, but Not Adjuvant Radiation Therapy, Improves Survival in Stage I and II Oral Tongue Cancer with Depth of Invasion >4 mm

**DOI:** 10.7759/cureus.6288

**Published:** 2019-12-04

**Authors:** Justin Mann, Diana Julie, Sean S Mahase, Debra D'Angelo, Louis Potters, A. Gabriella Wernicke, Bhupesh Parashar

**Affiliations:** 1 Radiation Oncology, Memorial Sloan Kettering Cancer Center, New York, USA; 2 Radiation Oncology, NewYork-Presbyterian/Weill Cornell Medical Center, New York, USA; 3 Biostatistics and Epidemiology, Weill Cornell Medical Center, New York, USA; 4 Radiation Oncology, Zucker School of Medicine at Hofstra/Northwell, New York, USA; 5 Radiation Oncology, Weill Cornell Medical Center, New York, USA

**Keywords:** depth, invasion, radiation, tongue, cancer, ncdb

## Abstract

Purpose/objective(s)

In early-stage, node negative oral tongue cancer, there is limited data supporting tumor depth of invasion (DOI) as an indication for post-operative radiotherapy (PORT) to the primary site. The primary aim of this study is to examine the effect of tumor DOI and PORT on overall survival (OS).

Materials and methods

The National Cancer Database (NCDB) was used to query patients with AJCC stage I and II oral tongue cancer (2006-2013). Patients were stratified by receipt of PORT, elective neck dissection (ND), and DOI (≤4 mm or >4 mm). Kaplan-Meier analysis was performed to compare OS (using the log-rank test) between PORT versus no-PORT. Multivariable Cox proportional hazards regression model performed to evaluate the independent effect of PORT and neck dissection on OS.

Results

Among 939 patients, 69.3% were clinical stage I, 67.4% received ND, 23.4% had DOI >4 mm, and 10.4% received PORT. The addition of PORT did not improve OS with tumor DOI ≤4 mm (p = 0.634) or >4 mm (p = 0.816). The addition of elective neck dissection improved OS for DOI >4 mm (p = 0.010), but not for ≤4 mm (p = 0.128). On multivariable analysis, ND improved OS if DOI >4 mm (HR, 0.37; 95% CI, 0.17-0.81 [p = .012]), when also controlling for age, sex, PORT status, clinical stage, and pathological stage.

Conclusion

Tumor DOI should not be used as a sole indication for PORT in early stage oral tongue cancers. Elective neck dissection at the time of excision of the primary tumor results in higher OS for tumors with DOI >4 mm.

## Introduction

Depth of invasion (DOI) is defined as the length measured from the tumor surface to the deepest point of invasive tumor in a paraffin embedded section [1]. The cut-off commonly used to stratify patients into low and high risk is 4 mm [2]. DOI is an important prognostic factor for nodal metastasis in oral tongue cancer, with increasing DOI associated with nodal involvement and worse prognosis [3-7].

Recent studies demonstrated no benefit to adding radiation therapy (RT) for deeper tumors [5-9]. O'steen et al. retrospectively evaluated the outcomes of 32 patients with stage N0-2b oral tongue or floor of mouth cancers with the primary tumor not crossing the midline who underwent PORT. The DOI in >75% patients was >4 mm, >75% had positive or close (<5 mm) margins and 38% had perineural invasion (PNI). RT to contralateral (CL) neck was omitted. At a median follow-up of 5.5 years among patients alive at the end of the study, there were no isolated nodal recurrences despite the majority of tumors possessing of DOI >4 mm. The authors concluded that the risk of nodal recurrence when omitting CL neck RT was very low if the primary tumor did not cross the midline, irrespective of other risk factors [5].

Rajappa et al. evaluated 375 pT1-2N0 oral tongue cancer patients. The cohort’s median age was 49, and 93% had squamous cell carcinomas, with 37.6% and 5.87% possessing PNI and lympho-vascular invasion (LVI), respectively. PORT was delivered in 37.6% of the cohort for PNI/LVI in the majority of cases, and for close margins in the remaining patients. At a mean follow-up of 40.9 months, there was a 18.4% local recurrence rate, with a 12.7-month mean duration of recurrence. Forty-four percent of the recurrences were salvaged while the remainder developed distant metastasis (DM) or unresectable disease. The two- and five-year overall survival (OS) were 94.5% and 93.9%, respectively. The patients were further divided into three groups: DOI <5 mm, 6-10 mm and >10 mm, each of which were categorized into RT vs no-RT groups. Adding RT did not improve OS or disease-free survival (DFS) in any group [8].

A National Cancer Database (NCDB) analysis of 934 patients with pathological T2N0 oral tongue cancers from 2004-2013 was performed to determine whether lesions with >5 mm DOI benefitted from receiving PORT [9]. Six hundred and seventy-seven (72.5%) patients had surgery alone and 257 (27.5%) received surgery plus PORT. Thirty-four (13.4%) received chemotherapy in addition to surgery and PORT. With a median follow-up of 28.4 months +/-10.4 months, the three-year OS was 81.3%. In multivariate analysis (MVA), adding PORT did not improve OS, even for patients with >5 mm DOI (p = 0.769).

This study evaluates the potential benefit of PORT in pT1-2N0 (stage I and II) oral tongue cancers with a DOI >4 mm.

## Materials and methods

The NCDB is a national oncology database and the data represents >70% newly diagnosed cancer cases and >34 million historical records [10]. This study was deemed to be exempt as per our Institutional review board. Patients with AJCC stage I and II oral tongue cancer diagnosed between 2006 and 2013 were queried. Inclusion criteria entailed oral cavity tumors (tongue) with wide excision, stage I and II, histology codes 8052, 8070-8078, and 8083. Patients receiving chemotherapy, had an OS less than six months, underwent any RT other than EBRT, or any residual tumor after surgical resection, were excluded from analysis.

**Figure 1:**
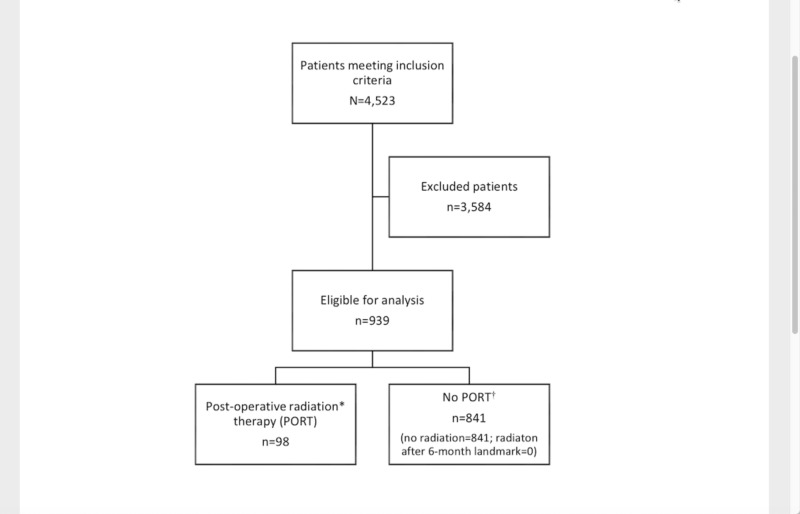
Patient inclusion flow diagram.

Patients were stratified by receipt of PORT, elective neck dissection, and extent of tumor DOI (≤4 mm or >4 mm). Kaplan-Meier analysis was performed to compare OS (using the log-rank test) between patients receiving and not receiving PORT. Multivariable Cox proportional hazards regression model was used to evaluate the independent effect of PORT on OS, while controlling for tumor DOI and other clinical characteristics. The patient inclusion flow diagram is shown in Figure 1.

## Results

Patient characteristics are summarized in Table 1 and comparisons of patients in the <4 mm DOI and >4 mm DOI groups are shown in Tables 2, 3. Ninety-eight (10.5%) patients received RT while 841 (89.5%) did not. Overall, a greater percentage of patients did not receive PORT. This trend persevered among each tumor stage. Additionally, African Americans were less likely to undergo PORT. In addition, the patients not receiving PORT were more likely to have LVI in the <4 mm category.

**Table 1:** Patient characteristics by post-operative radiation therapy (PORT) status. *All continuous variables were analyzed using the Wilcoxon rank sum test, and all categorical variables were analyzed using the Chi-squared test, except those denoted with †, in which Fisher’s exact test was used.

	PORT			No PORT	
Characteristic	N	Mean (SD) or Freq. (%)	N	Mean (SD) or Freq. (%)	p-value*
Age at Diagnosis	98	57.8 (14.0)	841	59.5 (15.0)	0.346
Sex	98		841		
Male	57	58.2%	443	52.7%	0.303
Female	41	41.8%	398	47.3%	
Race	98		841		
Black	7	7.1%	15	1.8%	0.015^†^
White	84	85.7%	780	92.8%	
Others	6	6.1%	36	4.3%	
Unknown	1	1.0%	10	1.2%	
Time from Diagnosis to Treatment (Days)	98	26.2 (19.0)	803	28.5 (31.9)	0.865
Time from Diagnosis to Surgery (Days)	98	28.1 (19.3)	803	33.9 (32.1)	0.124
Time from Diagnosis to Radiation Therapy (Days)	98	79.0 (27.8)	0	--	--
Tumor Grade	98		841		
Well differentiated, differentiated, NOS	23	23.5%	275	32.7%	0.124
Moderately differentiated, moderately well differentiated, intermediate differentiation	56	57.1%	435	51.7%	
Poorly differentiated	15	15.3%	83	9.9%	
Cell type not determined, not stated or not applicable, unknown primaries, high-grade dysplasia	4	4.1%	48	5.7%	
Clinical Stage	98		841		
Stage I	39	39.8%	612	72.8%	<0.001
Stage II	59	60.2%	229	27.2%	
Pathological Stage	98		841		
Stage I	50	51.0%	670	79.7%	<0.001
Stage II	48	49.0%	171	20.3%	
Analytic Stage	98		841		
Stage I	50	51.0%	670	79.7%	<0.001
Stage II	48	49.0%	171	20.3%	
Tumor Depth	98		841		
≤4 mm	62	63.3%	657	78.1%	0.001
>4 mm	36	36.7%	184	21.9%	
Regional Lymph Node Surgery	98		841		
Yes	80	81.6%	553	65.8%	0.002
No	18	18.4%	288	34.2%	
Lymph Vascular Invasion	93		813		
Present	12	12.9%	31	3.8%	0.006^†^
Not present	70	75.3%	681	83.8%	
Not applicable	1	1.1%	9	1.1%	
Unknown	10	10.8%	92	11.3%	

**Table 2:** Characteristics of patients with tumor depth ≤4 mm by post-operative radiation therapy (PORT) status. *All continuous variables were analyzed using the Wilcoxon rank sum test, and all categorical variables were analyzed using the Chi-squared test, except those denoted with †, in which Fisher’s exact test was used.

	PORT			No PORT	
Characteristic	N	Mean (SD) or Freq. (%)	N	Mean (SD) or Freq. (%)	p-value*
Age at Diagnosis	62	56.9 (14.0)	657	59.6 (15.1)	0.158
Sex	62		657		
Male	35	56.5%	343	52.2%	0.522
Female	27	43.6%	314	47.8%	
Race	62		657		
Black	3	4.8%	11	1.7%	0.273^†^
White	56	90.3%	609	92.7%	
Others	2	3.2%	29	4.4%	
Unknown	1	1.6%	8	1.2%	
Time from Diagnosis to Treatment (Days)	62	28.0 (19.9)	657	28.9 (34.4)	0.435
Time from Diagnosis to Surgery (Days)	62	29.3 (18.8)	657	34.9 (34.6)	0.360
Time from Diagnosis to Radiation Therapy (Days)	62	78.4 (28.2)	0	--	--
Tumor Grade	62		657		
Well differentiated, differentiated, NOS	16	25.8%	233	35.5%	0.078
Moderately differentiated, moderately well differentiated, intermediate differentiation	33	53.2%	320	48.7%	
Poorly differentiated	11	17.7%	61	9.3%	
Cell type not determined, not stated or not applicable, unknown primaries, high-grade dysplasia	2	3.2%	43	6.5%	
Clinical Stage	62		657		
Stage I	25	40.3%	502	76.4%	<0.001
Stage II	37	59.7%	155	23.6%	
Pathological Stage	62		657		
Stage I	33	53.2%	538	81.9%	<0.001
Stage II	29	46.8%	119	18.1%	
Analytic Stage	62		657		
Stage I	33	53.2%	538	81.9%	<0.001
Stage II	29	46.8%	119	18.1%	
Regional Lymph Node Surgery	62		657		
Yes	52	83.9%	402	61.2%	<0.001
No	10	16.1%	255	38.8%	
Lymph Vascular Invasion	58		631		
Present	8	13.8%	19	3.0%	0.005^†^
Not present	44	75.9%	528	83.7%	
Not applicable	1	1.7%	9	1.4%	
Unknown	5	8.6%	75	11.9%	

**Table 3:** Characteristics of patients with tumor depth >4 mm by post-operative radiation therapy (PORT) status. *All continuous variables were analyzed using the Wilcoxon rank sum test, and all categorical variables were analyzed using the Chi-squared test, except those denoted with †, in which Fisher’s exact test was used.

	PORT			No PORT	
Characteristic	N	Mean (SD) or Freq. (%)	N	Mean (SD) or Freq. (%)	p-value*
Age at Diagnosis	36	59.3 (14.1)	184	59.1 (14.8)	0.704
Sex	36		184		
Male	22	61.1%	100	54.4%	0.455
Female	14	38.9%	84	45.7%	
Race	36		184		
Black	4	11.1%	4	2.2%	0.014^†^
White	28	77.8%	171	92.9%	
Others	4	11.1%	7	3.8%	
Unknown	0	0%	2	1.1%	
Time from Diagnosis to Treatment (Days)	36	23.2 (17.3)	175	26.9 (20.5)	0.397
Time from Diagnosis to Surgery (Days)	36	26.0 (20.2)	175	30.0 (20.9)	0.284
Time from Diagnosis to Radiation Therapy (Days)	36	79.9 (27.5)	0	--	--
Tumor Grade	36		184		
Well differentiated, differentiated, NOS	7	19.4%	42	22.8%	0.763^†^
Moderately differentiated, moderately well differentiated, intermediate differentiation	23	63.9%	115	62.5%	
Poorly differentiated	4	11.1%	22	12.0%	
Cell type not determined, not stated or not applicable, unknown primaries, high-grade dysplasia	2	5.6%	5	2.7%	
Clinical Stage	36		184		
Stage I	14	38.9%	110	59.8%	0.021
Stage II	22	61.1%	74	40.2%	
Pathological Stage	36		184		
Stage I	17	47.2%	132	71.7%	0.004
Stage II	19	52.8%	52	28.3%	
Analytic Stage	36		184		
Stage I	17	47.2%	132	71.7%	0.004
Stage II	19	52.8%	52	28.3%	
Regional Lymph Node Surgery	36		184		
Yes	28	77.8%	151	82.1%	0.546
No	8	22.2%	33	17.9%	
Lymph Vascular Invasion	35		182		
Present	4	11.4%	12	6.6%	--
Not present	26	74.3%	153	84.1%	
Not applicable	0	0%	0	0%	
Unknown	5	14.3%	17	9.3%	

**Figure 2:**
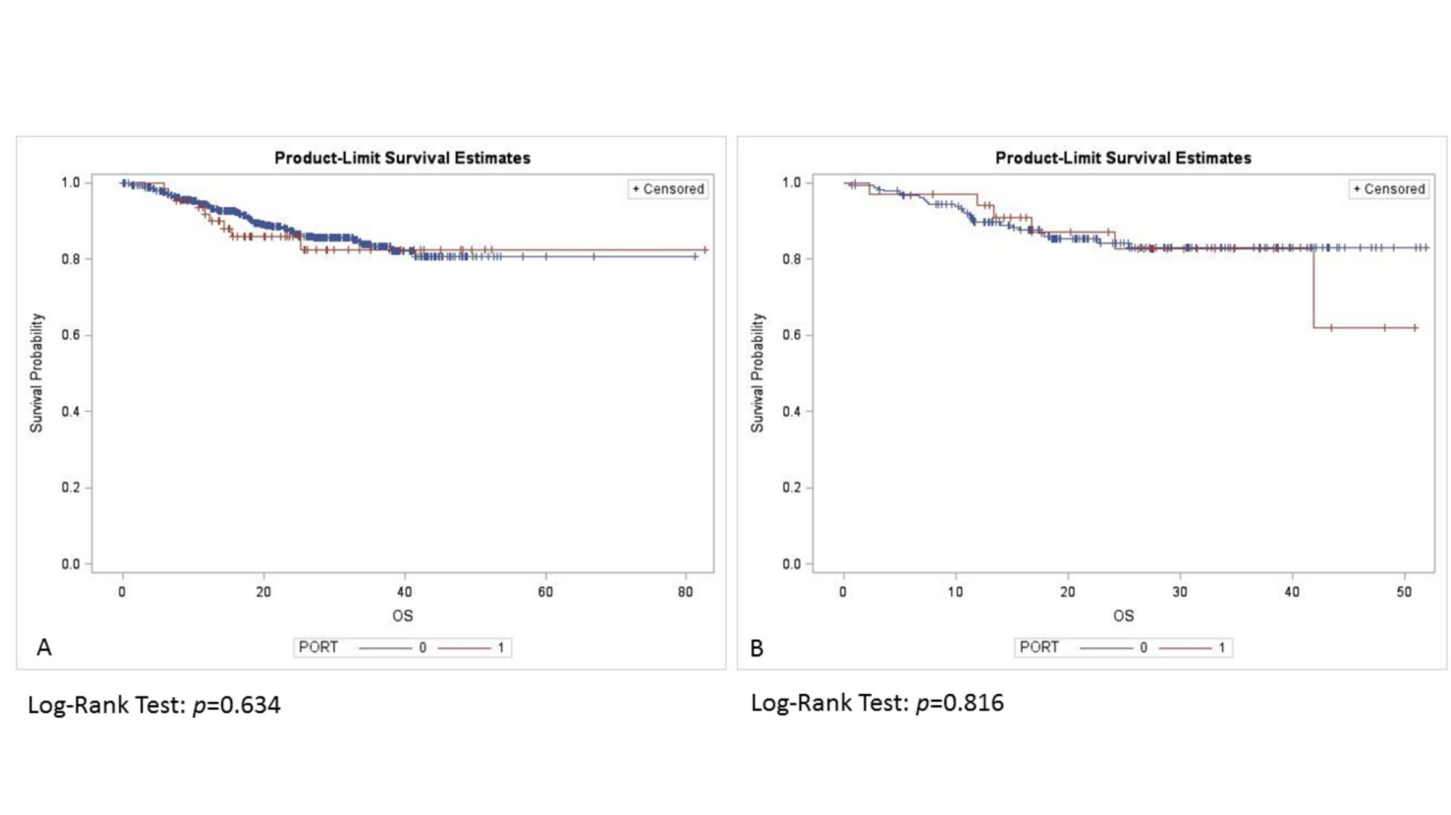
Overall survival by PORT status with DOI: (A) <4 mm, (B) >4 mm. PORT: Post-operative radiotherapy; DOI: Depth of invasion.

**Figure 3:**
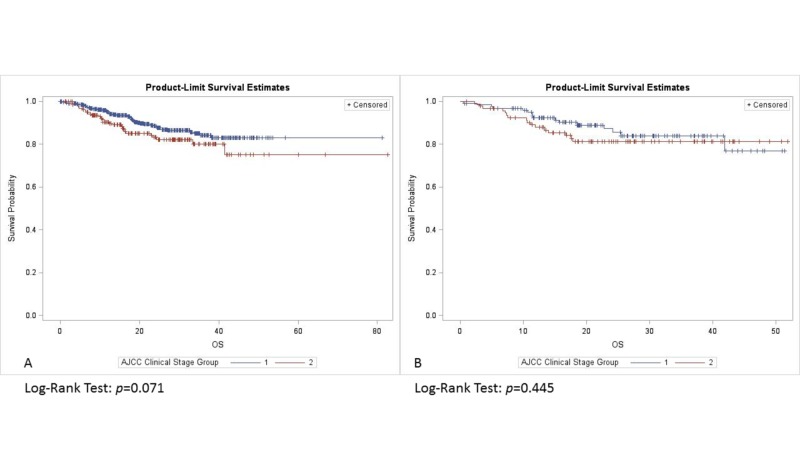
Overall survival by clinical stage with DOI: (A) <4 mm, (B) >4 mm. DOI: Depth of invasion

**Figure 4:**
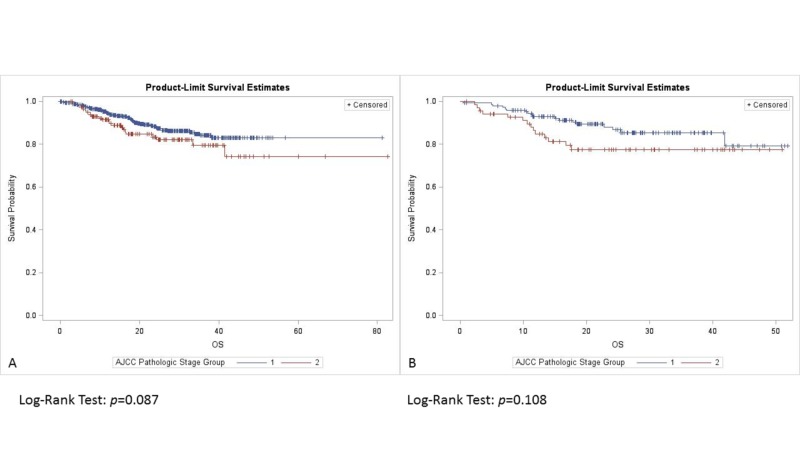
Overall survival by pathological stage with DOI: (A) <4 mm, (B) >4 mm. DOI: Depth of invasion

For tumors <4 mm DOI, adding RT did not improve survival (p = 0.634). OS was similar in patients with DOI >4 mm with or without RT (p = 0.816) (Figure 2). Among those with tumor DOI <4 mm, clinical stage I patients trended towards improved OS compared to patients with clinical stage II tumors (p = 0.07), and those with pathological stage I tumors trended towards improved OS in comparison to pathological stage II lesions (p = 0.087). There was no difference in OS with respect to clinical stage (p = 0.445) (Figure 3), and pathological stage (p = 0.108) (Figure 4) among patients with a tumor DOI >4 mm.

**Figure 5:**
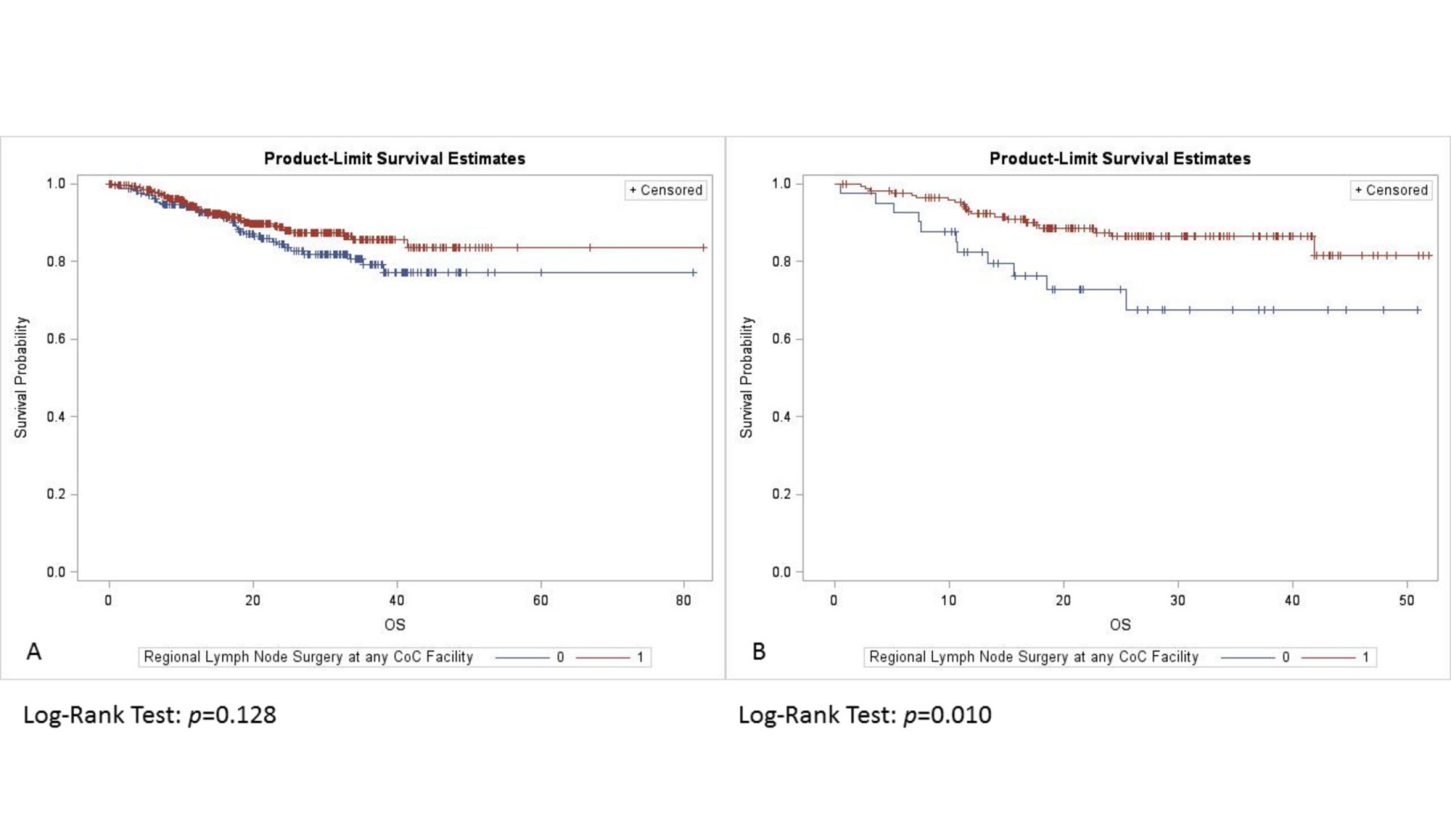
Overall survival by elective neck surgery for tumors with DOI: (A) <4 mm, (B) >4 mm. DOI: Depth of invasion

While elective neck dissection (END) did not impact OS for lesions with DOI <4 mm (p = 0.128), it did confer a survival benefit for lesions with DOI >4 mm (p = 0.01) (Figure 5).

On multivariable survival analysis, END remained associated with an improved OS in the subset of patients with a DOI >4 mm (hazard ratio of death, 0.37; 95% confidence interval, 0.17-0.81 [p = 0.012]), when also controlling for age, sex, PORT status, clinical stage, and pathological stage (Tables 4, 5).

**Table 4:** Multivariable Cox regression model for overall survival (OS) tumor depth ≤4 mm. PORT: Post-operative radiotherapy

Characteristic	Hazard Ratio (95% CI)	p-Value
PORT		
No	--	--
Yes	1.11 (0.54, 2.27)	0.782
Age	1.03 (1.01, 1.05)	<0.001
Sex		
Male	--	--
Female	0.84 (0.54, 1.30)	0.425
Clinical Stage		
Stage I	--	--
Stage II	1.55 (0.83, 2.90)	0.172
Pathological Stage		
Stage I	--	--
Stage II	1.27 (0.66, 2.45)	0.482
Regional Lymph Node Surgery		
No	--	--
Yes	0.72 (0.45, 1.14)	0.159

**Table 5:** Multivariable Cox regression model for overall survival (OS) tumor depth >4 mm. PORT: Post-operative radiotherapy

Characteristic	Hazard Ratio (95% CI)	p-Value
PORT		
No	--	--
Yes	0.80 (0.32, 2.01)	0.641
Age	1.04 (1.01, 1.07)	0.011
Sex		
Male	--	--
Female	0.69 (0.33, 1.44)	0.319
Clinical Stage		
Stage I	--	--
Stage II	1.03 (0.42, 2.57)	0.943
Pathological Stage		
Stage I	--	--
Stage II	2.00 (0.82, 4.89)	0.129
Regional Lymph Node Surgery		
No	--	--
Yes	0.37 (0.17, 0.81)	

## Discussion

This population-based study of stage I and II oral tongue cancers showed a survival benefit of elective neck dissection in patients with DOI >4 mm, but no benefit of adding adjuvant RT in regard of less of DOI.

Numerous studies identify DOI as a poor prognostic factor. A retrospective Japanese study of 337 stage I-II tongue cancer patients undergoing surgical resection revealed that T stage, DOI (cut-off was 4 mm), tumor budding (the presence of a single cancer cell or cluster of less than five cancer cells at the invasive front) and adjacent tissue at the invasive front are predictive of delayed neck metastasis [11].

Although 4 mm is commonly considered the DOI cut off for significance, a retrospective study of DOI cut-off points in previously untreated early stage oral tongue cancers showed 7.25 mm to be most predictive of occult nodal metastasis, 8 mm for OS and DFS [3]. In another retrospective study of 93 early stage oral lung cancer patients undergoing primary resection without neck dissection, 47.4% had nodal recurrence, with 19.7% recurred at the primary site. Cox-proportional polynomial analysis showed an increasing hazard of recurrence with DOI between 2-6 mm [4].

Ganly et al. sought to determine factors associated with tumor recurrence in a cohort of 216 patients with oral tongue cancers. Half of the lesions were T2, 83% underwent surgery and 17% underwent surgery and PORT. At a median follow-up of 80 months, MVA revealed DOI as an independent predictor of neck relapse-free survival, with a DOI >2 mm conferring 3.7-fold higher risk of recurrence compared to DOI <2 mm [12].

A retrospective review evaluated outcomes of 103 patients with T1 or T2 N0 oral tongue cancers who underwent surgical resection with negative margins and DOI >4 mm. Sixty-two patients received PORT and 41 did not. With a median follow-up of 41.3 months, there was no difference between PORT versus no PORT [13].

Shim et al. reviewed the medical records of 86 patients with oral tongue cancers, of which 58% were stage I, 26% stage II and 16% stage III. Among the 16% receiving PORT, they reported no difference in recurrence rates for tumors >0.5 cm compared to those who did not receive PORT [14]. Table 6 summarizes select studies evaluating DOI as a prognostic factor.

**Table 6:** Select studies evaluating DOI as a prognostic factor. DOI: Depth of invasion; MVA: Multivariate analysis; LVI: Lympho-vascular invasion; PNI: Perineural invasion; HPV: Human papillomavirus.

Author, year (reference)	Study design	Significant DOI	Outcomes	Comments
Fukano et al., 1997 [15]	Retrospective, 34 patients, oral tongue cancer	5 mm	>5 mm, neck metastasis 64.7%	For DOI > 5 mm, suggestion is to operate or radiate neck
Asakage et al., 1998 [16]	Retrospective, 44 patients, oral tongue, stage I/II partial glossectomy only	4 mm	Cervical metastasis in 21/44 patients, >4 mm only factor significant in MVA	Recommended supraomohyoid neck dissection in tumors > 4 mm.
Kurokawa et al., 2002 [17]	Retrospective, 50 patients, stage I/II oral tongue, only partial glossectomy	4 mm	Overall cervical metastasis rate of 14%, MVA showed DOI > 4 mm as the significant risk factor	Recommended to electively treat the neck for DOI > 4 mm
Goodman et al., 2009 [18]	SEER, DOI, LVI and PNI assessed with respect to mortality	3 mm	MVI showed DOI and PNI were significant predictors of OS	
Ling et al., 2013 [19]	Retrospective, 210 patients with tongue cancer	9 mm	DOI > 9 mm 7.7 times more likely to die than tumors <4 mm	To improve survival in such patients, surgical resection recommended.
Almangush et al., 2014 [20]	Retrospective study of 233 patients with stage I/II oral tongue cancers	4 mm	Tumor budding and DOI > 4 mm associated with worse prognosis	Recommended multimodality therapy for deep tumors.
Masood et al., 2018 [21]	Retrospective study, 67 patients with T1/2N0 oral tongue cancer HPV-	5 mm	DOI > 5 mm associated with risk of LVI and nodal metastasis	No specific recommendation made regarding management.

Limitations of our study include its retrospective nature, relatively small number in the total group receiving RT and lack of data on details of treatment such as technique of RT, use of image guidance and dose, local control and toxicity. Select studies evaluating DOI as a prognostic factor are listed in Table 6. In clinical practice, DOI does dictate neck dissection based on risk of neck metastasis although we show survival benefit with END in DOI > 4 mm. RT is associated with significant side effects including mucositis, pain, dysphagia, necrosis, dry mouth and loss of taste, and can be avoided for early stage tongue cancers.

## Conclusions

Our study is the first large population-based study of both stage I and II oral cavity cancers to show addition of elective neck irradiation for tumors >4 mm does not improve survival. However, elective neck dissection in oral tongue cancers with DOI >4 mm confers a positive survival benefit.
